# Effect of systemic atorvastatin on bone regeneration in critical-sized defects in hyperlipidemia: an experimental study

**DOI:** 10.1186/s40729-023-00508-9

**Published:** 2023-12-14

**Authors:** Kübra Öztürk, Turan Emre Kuzu, Semih Ayrıkçil, Cem Abdulkadir Gürgan, Gözde Özge Önder, Arzu Yay

**Affiliations:** 1 Department of Oral and Maxillofacial Surgery, Faculty of Dentistry, Nuh Naci Yazgan University, Kayseri, Türkiye; 2 Department of Periodontology, Faculty of Dentistry, Nuh Naci Yazgan University, Kayseri, Türkiye; 3 Department of Histology and Embryology, Faculty of Medicine, Erciyes University, Kayseri, Türkiye

**Keywords:** Hyperlipidemia, Xenograft, Atorvastatin, Bone regeneration

## Abstract

**Purpose:**

Hypocholesterolemic medications similar to atorvastatin are efficient in lowering blood lipid levels; however, compared to other medications in the statin family, their impact on bone metabolism is claimed to be insufficient. The impact of atorvastatin on bone regeneration in dental implantology in individuals with hyperlipidemia who received atorvastatin in the clinic is doubtful.

**Methods:**

In the study, 16 male New Zealand rabbits of 6 months were used. All rabbits were fed a high-cholesterol diet for 8 weeks, and hyperlipidemia was created. It was confirmed that the total cholesterol level in rabbits was above 105 mg/dl. A critical-sized defect was created in the mandible. The defect was closed with xenograft and membrane. Oral 10 mg/kg atorvastatin was started in the experimental group, and no drug was administered in the control group. At 16th week, animals were sacrificed. For histomorphological examination, the new bone area, osteoclast, and osteoblast activities were evaluated.

**Results:**

While new bone area (45,924 µm^2^, *p* < 0.001) and AP intensities (105.645 ± 16.727, *p* = 0.006) were higher in the atorvastatin group than in the control group, TRAP intensities in the control group (82.192 ± 5.346, *p* = 0.021) were higher than that in the atorvastatin group.

**Conclusions:**

It has been found that high blood lipid levels will adversely affect bone graft healing and the use of systemic atorvastatin contributes to bone healing. Clinicians should pay attention to the selection of surgical materials, considering the importance of questioning drug use in their patients and the risks in cases of non-use.

## Background

Hyperlipidemia is characterized by an abnormal lipid profile with high triglyceride (TG), total cholesterol, and low-density lipoprotein (LDL) levels, and lower-than-normal levels of high-density lipoprotein (HDL) cholesterol are known as hyperlipidemia [1]. Metabolic alterations, such as poor bone mineral density, an increase in the number of osteoclasts, and suppression of osteoblastic activity, have been shown to be responsible for the effect of hyperlipidemia on bone tissue metabolism [1, 2].

Statins are frequently administered as hypocholesterolemic medications for the treatment of heart diseases [3]. This group of drugs reversibly inhibits 3-hydroxy-3-methyl glutaryl coenzyme A (HMG-CoA) reductase, which plays an important role in cholesterol biosynthesis; lowers plasma cholesterol, LDL, apolipoprotein-B, and TGs; and increases HDL levels [4]. Owing to variations in their polarity and bone availability, the statin group of medications exhibits structural variances among its members, such as lipophilic or hydrophilic [5]. For instance, some statins, such as pravastatin, atorvastatin, and fluvastatin, are more hydrophilic than others, such as simvastatin and lovastatin, which are lipophilic [5, 6]. Statins have been found to affect bone tissue and promote the expression of bone morphogenetic proteins (BMP). While hydrophilic statins, such as atorvastatin and fluvastatin, have adverse effects on bone metabolism, lipophilic simvastatin is recognized to be helpful for bone mineral density and bone markers [5].

In recent years, speculative statements have been made in both visual and written communication organs that cholesterol is not an etiological factor for many cardiovascular diseases, particularly atherosclerosis. In a study by Dincer et al., the proportion of patients who were affected by the media and discontinued statin therapy was 52.9% [7]. However, statin group drugs are the first to come to mind in the picture of hypercholesterolemia and are commonly used in clinical practice. The current study began with the question of the effect of atorvastatin, which is used to improve the picture of hyperlipidemia, on bone metabolism.

The aim of this study is to investigate the impact of systemic atorvastatin administration in hypercholesterolemic rabbits on new bone formation in bone grafting procedures. This study aims to compare the effects of atorvastatin with hypercholesterolemic rabbits not receiving medication as the control group.

## Methods

### Animals

This study was approved by the Ethics Committee of Erciyes University Animal Experiments (19/173). Sixteen male New Zealand rabbits (6 months), with an average weight of 2–2.5 kg were included in the study. Animals were housed in individual cages with 12-h day/night cycles at 21 °C and free access to food and water.

### Induction of hyperlipidemia and experimental procedures

**Fig. 1:**
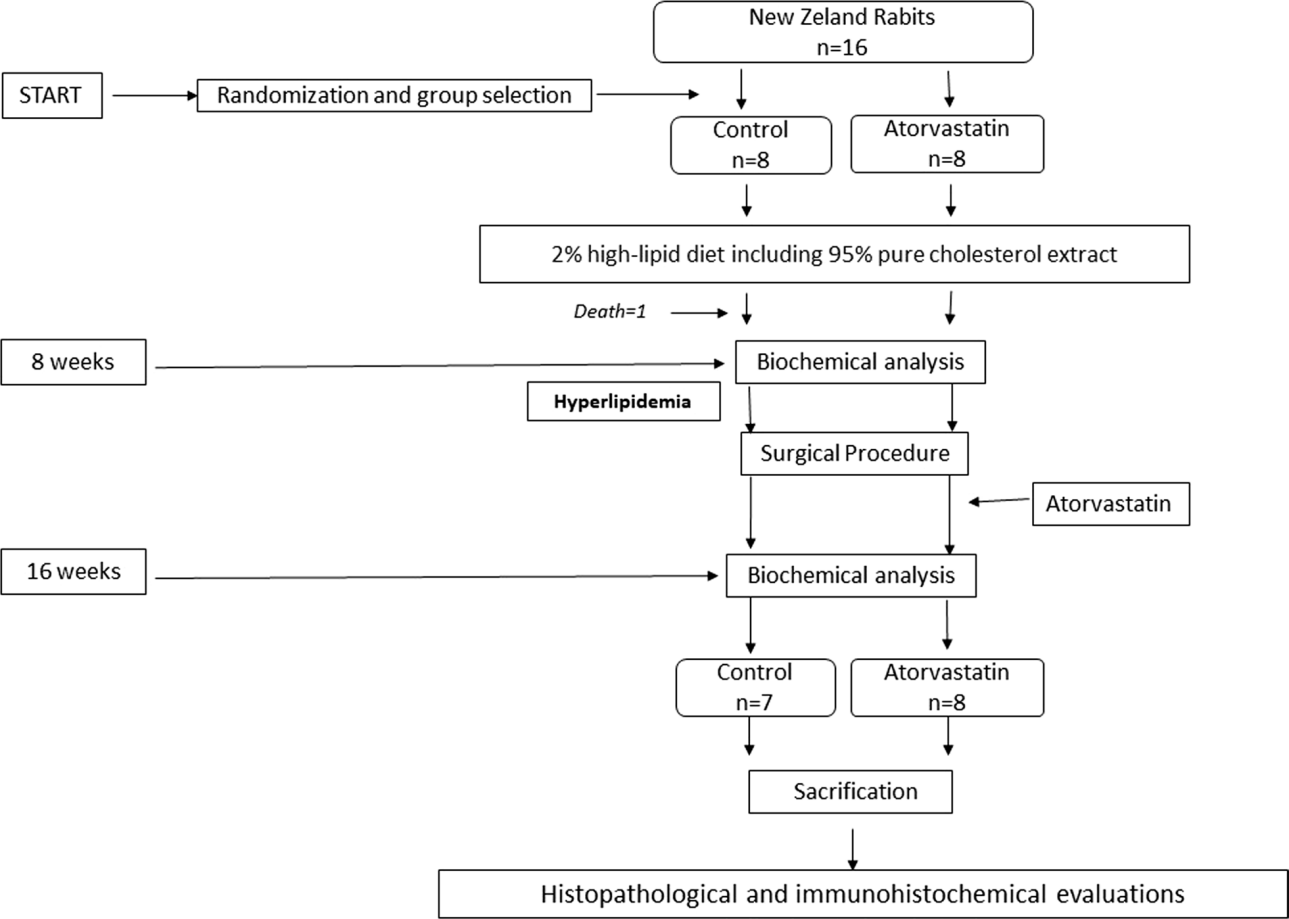
Flowchart of the study

We used simple randomization method; consequently, random numbers were generated using the standard (RAND) function in Microsoft Excel (Version 2211) after 1 week of adaptation. Each cage was numbered and followed-up. All animals were fed a 95% pure cholesterol and 2% high-fat diet for 8 weeks. At the end of 8 weeks, hyperlipidemia was observed in the blood taken from the ear veins, where all subjects were hyperlipidemic by biochemical methods. This was then passed on to the surgical stage. After the surgical phase was completed, the second group was administered 10 mg/kg atorvastatin (Alvastin; Aris, Türkiye) daily. In the 16th week, blood was drawn again from both groups to control hyperlipidemia. Triglyceride, total cholesterol, high-density lipoprotein (HDL), and low-density lipoprotein (LDL) levels were measured in blood at weeks 8 and 16 (Fig. 1).

### Surgical procedure

**Fig. 2:**
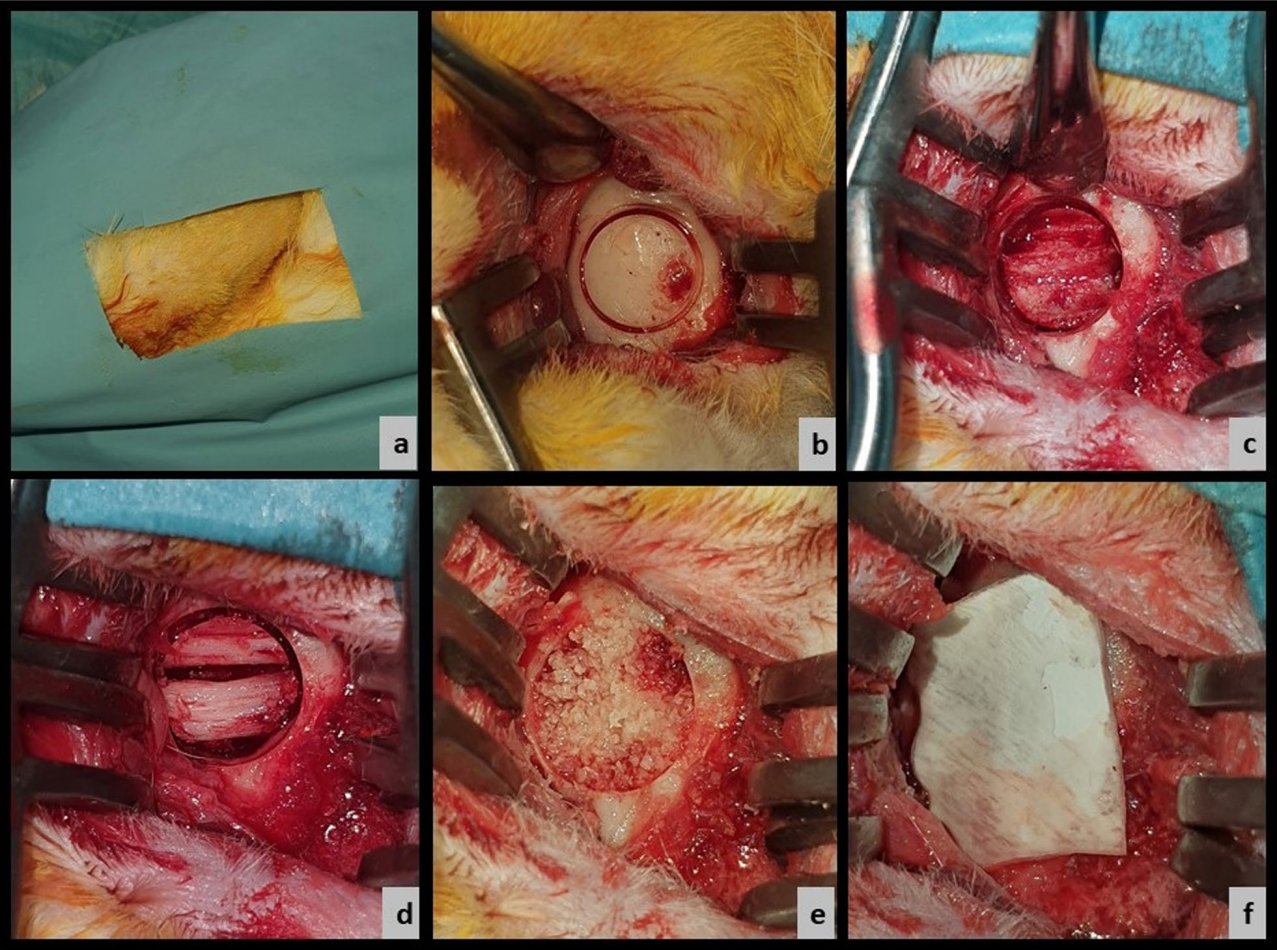
Surgical stages. **a** Surgical site preparation, **b** reaching the mandible and milling the bone, **c** tooth roots that are reached when the cortical bone is removed, **d** image of the defect after removing the roots, **e** filling the defect with xenograft, **f** closure of the defect with a collagen membrane

All animals were anesthetized by intramuscular injection of xylazine 2% (Rompun; Bayer, Leverkusen, Germany) and 40 mg/kg ketamine hydrochloride (Ketasol 10%; Richter Pharma Wels, Austria). The left mandibular corpus area was shaved, and the surgical site was cleaned with iodine solution. The surgical area was then covered with sterile drapes. One milliliter of local anesthetic solution (Ultracain D-S forte, Sanofi Aventis, Türkiye) was used to control the bleeding. A full-thickness incision was made, including the periosteum, with a length of 1.5 cm, and a number 15 scalpel. Anterior dissection of the masseter muscle was performed and the bone surface was exposed. Bone osteotomy was performed under sterile saline cooling with a 1 cm diameter and 5 mm deep trephine bur at the level of the corpus at the alignment of the 1st and 2nd molar teeth. The remaining midline bone was removed, and the roots were exposed. The roots of both the molars were removed, because they were within 5 mm of the depth. The defect was curetted to eliminate the periodontal ligaments. The defect was augmented with a bovine xenogeneic bone graft (Tutobone Microchips 0.25–1 mm, Tutogen Medical GmbH, Neunkirchen a. Brand, Germany) and a collagenous membrane derived from solvent preserved irradiated bovine pericardium (Tutopatch, Tutogen Medical GmbH, Neunkirchen a. Brand, Germany). First, the muscle, subcutaneous, and skin tissues were closed using 4.0 resorbable sutures (Pegelak, poly [glycolide-co-lactide]; Dogsan, Türkiye) (Fig. 2).

For postoperative care, ceftriaxone (40 mg/kg; Rocephin, Istanbul, Türkiye) and carprofen (3 mg/kg; Rimadyl; Pfizer, New York, USA) were administered intramuscularly once a day for 3 days.

After 16 weeks, the rabbits were euthanized with an overdose of 10% sevoflurane (Sevorane Liquid 100%, 250 mL solution).

### Histomorphometric analysis

**Fig. 3:**
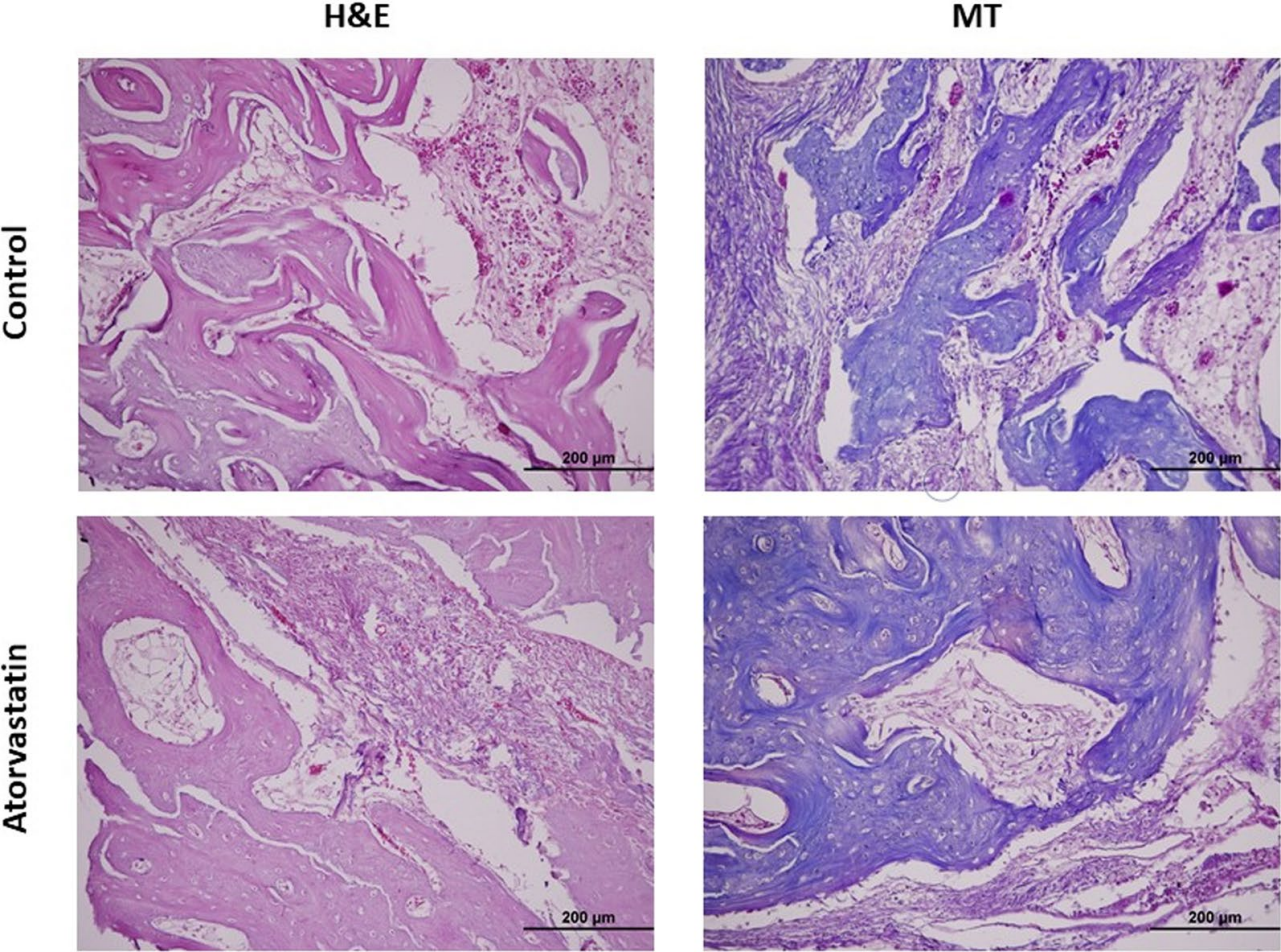
Histological sections in which the new bone area was evaluated by H&E (hematoxylin–eosin) and MT (Masson-trichrome) staining method. Original size, × 20

The blocks of the defective region with intact bone borders were removed. The samples were preserved in a 10% formaldehyde solution (pH 7) for 72 h before being rinsed under running water for histopathological analysis. The materials were decalcified in a regulated manner using an acidic approach (formaldehyde and nitric acid). The tissues were blocked using customary tissue follow-up and embedded in paraffin after fixation and decalcification. Using a microtome, 5 m slices of paraffin blocks prepared for light microscopic inspection were collected (Leica RM 2155). Slices (5 µm) cut from paraffin blocks were examined using Masson's trichrome (MT) and hematoxylin and eosin (H&E) staining. The sections were assessed by two blinded observers using a light microscope (BX51; Olympus, Tokyo, Japan). A total of five microscopic fields in each preparation of each group were photographed using a 20X lens to assess the ossification regions in the experimental groups. Using the ImageJ program (ImageJ, National Institute of Health, USA), newly formed bone areas (NBF) were computed, along with numerical values in square micrometers (Fig. 3).

### Immunohistochemical analysis

**Fig. 4:**
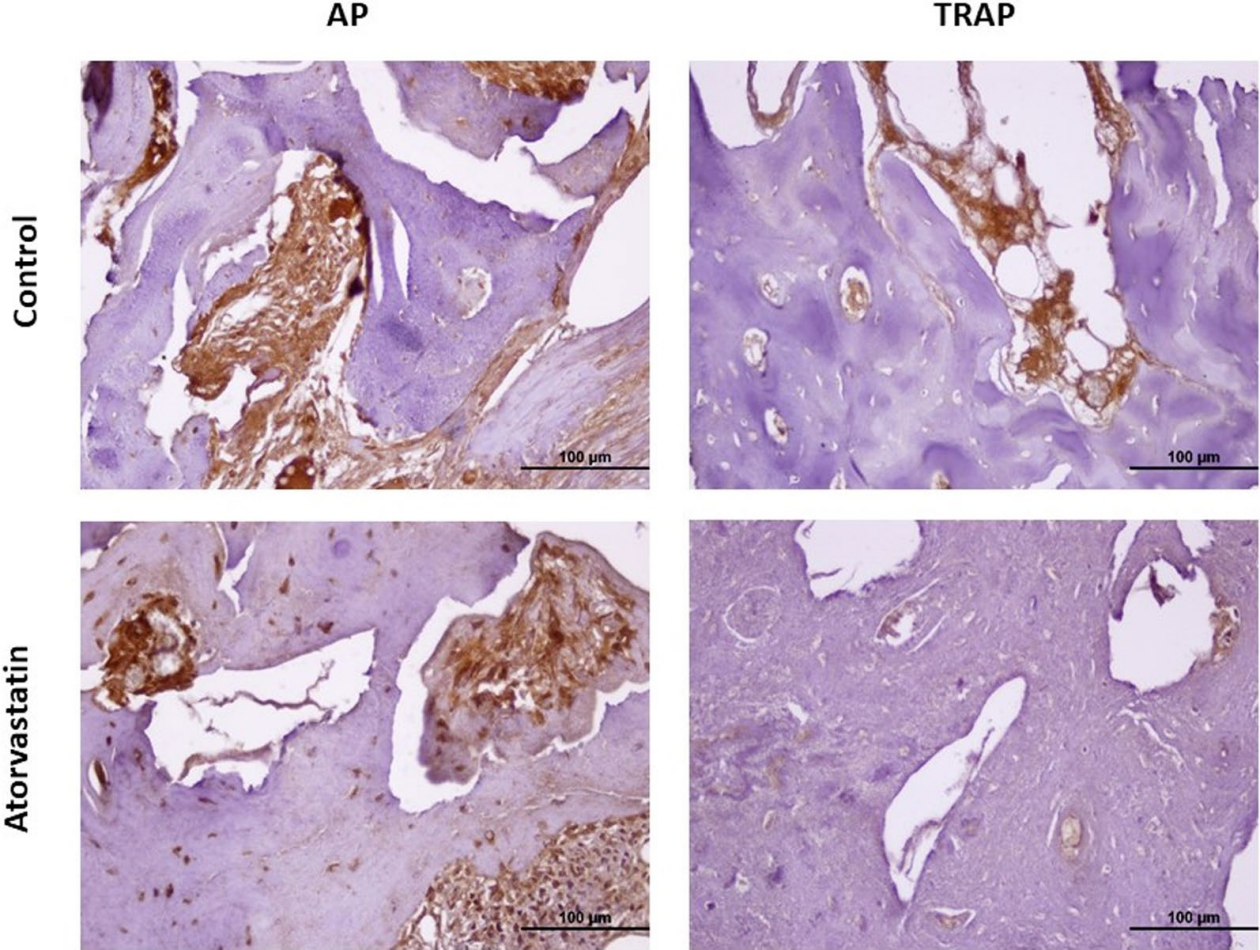
Immunohistochemical staining images of the study groups. TRAP staining images for quantitative assessment of osteoclast activity, AP staining images for quantitative assessment of osteoblast activity. *Original size, × 40

Avidin–biotin–peroxidase and immunohistochemical staining were used to assess the expression of TRAP (rabbit anti-TRAP/tartrate-resistant acid phosphatase polyclonal antibody, Abcam: bs-6434R, Cambridge, UK) and AP (Anti-Alkaline Phosphatase Antibody, Abcam: ab95462, Cambridge, UK) in all samples. A graded alcohol series and xylene were used to rehydrate 5 m pieces of paraffin blocks, which were subsequently rinsed three times for 5 min with phosphate buffer (PBS). The mixture was microwaved at 600 W for 5 min in 10% citrate buffer before cooling for 10 min at room temperature. After the second PBS wash, the sections underwent a 12-min treatment with 3% hydrogen peroxide (H_2_O_2_) to decrease endogenous peroxidase activity. The next stage involved the use of a staining kit for a large-volume detection system (Thermo Scientific, TP125-HL). To ensure that the portions outside the antigenic areas were covered, an ultra-V block was applied to the sections before rinsing with PBS once more for 5 min at room temperature. The sections were then immediately incubated with TRAP (Abcam: bs-6434R, Cambridge, UK) and AP (Abcam: ab95462, Cambridge, UK) primary antibodies before being stored at + 4 °C for one night and at room temperature for 30 min the following morning. After washing, the sections were incubated for 10 min with a biotin-secondary antibody before the washing procedure. After washing, the sections that had been exposed to streptavidin peroxidase for 10 min were then treated for 1–5 min with the peroxidase substrate from the kit with diaminobenzidine (DAB) characteristics, allowing the immunoreactivities to become apparent. Sections were rinsed several times with distilled water before counterstaining with Mayer's hematoxylin. The slices were then sealed with a closure solution (Entellan®, Merck), passed through xylene by eliminating the water with an increasing alcohol series, and inspected under a microscope (Olympus BX-51, Tokyo, Japan). The image J software program (ImageJ, National Institute of Health, USA) was used to calculate TRAP and AP immunoreactivity intensity in the lower jaw sections stained with immunohistochemical staining. Light microscopic photographs of each tissue were taken from five different regions at 40X magnification were used (Fig. 4). The immunoreactivity intensity was determined as the mean of the intensities using Image J software (NIH, USA) [8, 9].

### Statistical analysis

Sample size was determined using the GPower program (latest ver. 3.1.9.7; Heinrich-Heine-Universität Düsseldorf, Düsseldorf, Germany). For the effect size, Türer et al.’s work was taken as reference [10]. In the effect size calculation applied for two independent groups, the smallest effect size was determined as 1.6. When *α* = 0.05 and power was calculated as 0.90, a total of 14 samples and seven rabbits per group were calculated. Due to the possibility of animal loss, one rabbit was added to each group and the study was started with a total of 16 animals.

All statistical analyses were evaluated using IBM SPSS 22.0 (Chicago IL) and *p* < 0.05 was considered statistically significant. The normal distribution of values was determined using Shapiro–Wilk's and *Q*–*Q* graphs.

If the evaluation values between the groups showed a normal distribution, two independent samples *t* test (Student’s *t* test) was used; if it did not show a normal distribution, it was evaluated using the Mann–Whitney *U* test.

This study was designed and performed by the ARRIVE guideline [11].

## Results

During the experiment, one rabbit from the control group was lost after 8 weeks of hyperlipidemia. At the end of the study, the feeding and muscle reflexes of all the remaining rabbits were normal in the examination performed before killed, and there were no signs of infection in the maxillofacial region or urine.

The results of the biochemical analyses at 8 and 16 weeks are presented in Table 1. During the study, it was observed that the medication was effective in reversing hyperlipidemia.

**Table 1:** Biochemical parameters of the study groups at weeks 8 and 16 (mean ± SD)

	Control (*n* = 7)		Atorvastatin (*n* = 8)	
	8 weeks	16 weeks	8 weeks	16 weeks
Total cholesterol (mg/dl)	1367.33 ± 308.127	1649.50 ± 238.835	1472.00 ± 240.028	178.71 ± 54.61
LDL (mg/dl)	1399.317 ± 219.1969	1389.417 ± 237.6019	1302.757 ± 171.2078	162.214 ± 23.4015
HDL (mg/dl)	79.80 ± 21.6869	71.417 ± 15.2772	69.586 ± 23.9535	71.057 ± 19.1827
Trigliserid (mg/dl)	162.67 ± 27.208	179.33 ± 16.729	151.14 ± 25.609	65.00 ± 6.429

Sections of tissue taken from rabbits belonging to all experimental groups were stained with H&E and MT and examined under a light microscope. It was noted that in all groups, the defect area was generally filled with connective tissue, and there were new bone formation areas in between. Fatty tissue was observed in connective tissue areas. The least ossification was observed in the control group. It was observed that the ossification areas were more prominent in the atorvastatin-treated group compared to the control group (Fig. 3).

**Fig. 5:**
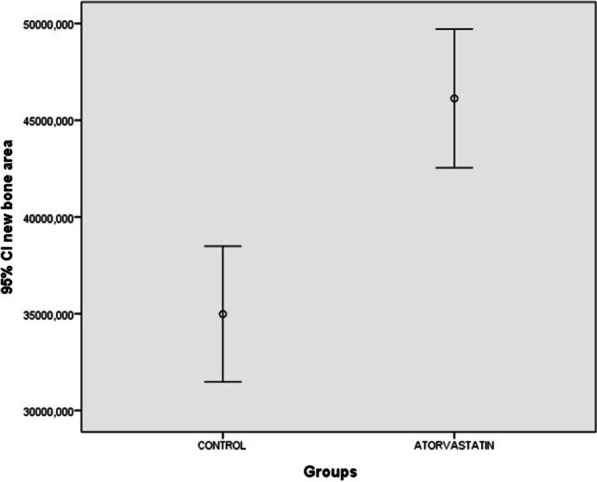
New bone area of study groups at 16 weeks (*p* < 0.001)

The lowest NBF was observed in the control group. The NBF was higher in the atorvastatin group (45924 µm^2^) than in the control group (29298 µm^2^) (*p* < 0.001) (Fig. 5).

**Fig. 6:**
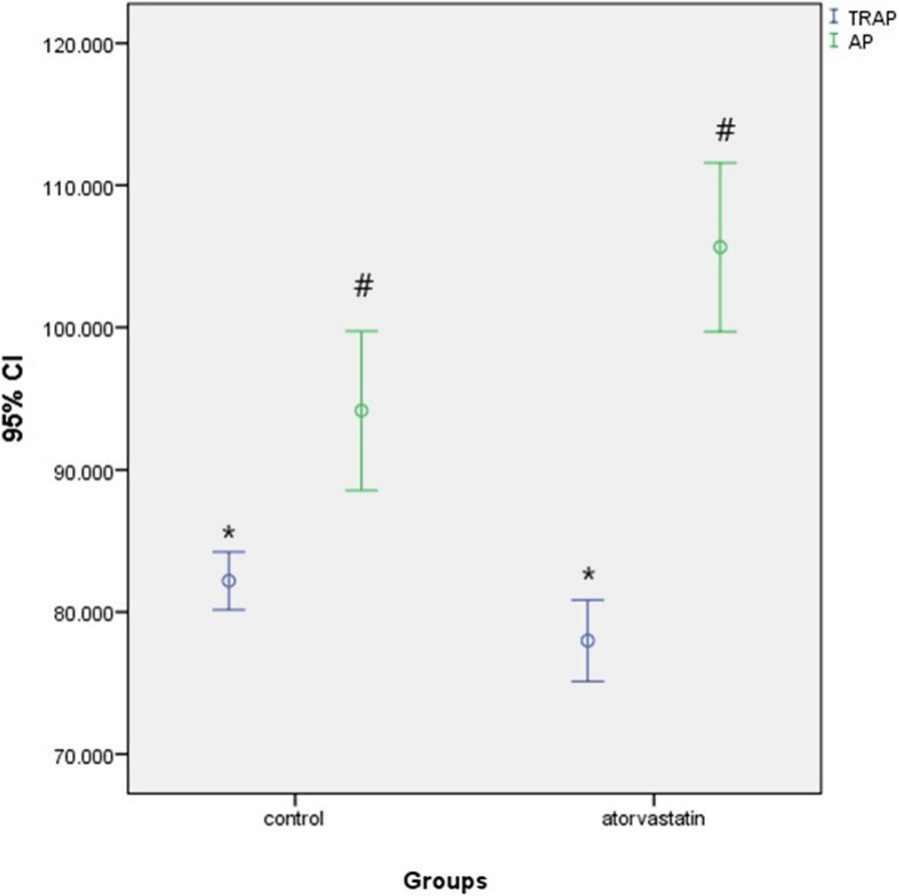
Comparison of TRAP and AP immunoreactivity between groups. *p = 0.021; *p* < 0.05 statistically significant and the control group is higher than the atorvastatin group. #*p* = 0.006; *p* < 0.05 statistically significant and the atorvastatin group is higher than the control group

Immunohistochemically, TRAP was used to determine osteoclast activity and AP was used to evaluate osteoblast activity. The cells were then examined under a light microscope. The mean TRAP values in the control and atorvastatin groups were 82.192 ± 5.346 and 77.989 ± 8.090, respectively. The difference between the groups was statistically significant (*p* = 0.021). The mean AP values were 94.145 ± 14.704 and 105.645 ± 16.727 for the control and atorvastatin groups, respectively. The difference between the groups was statistically significant (*p* = 0.006) (Fig. 6).

## Discussion

This study was conducted to determine the effect of atorvastatin on bone regeneration and investigate whether regeneration is negatively affected in patients who refuse to use this drug. According to the results of this study, atorvastatin prevented the negative effects of hypercholesterolemia on regeneration of histomorphometric and immunohistochemical parameters. This study also showed that atorvastatin did not contribute to bone metabolism despite its positive effect on the blood lipid profile but was ineffective in new bone metabolism compared to other statins. When the drug is administered systemically, it positively affects regeneration by acting on the mandible, and this improvement is clinically sufficient.

LDL, HDL, and very low-density lipoprotein (VLDL) are blood lipid proteins that affect regeneration in various ways [12]. According to Dündar et al., rabbits fed either a regular diet or a high-fat diet for 3 months showed no change in bone-implant contact 12 weeks after implant implantation [12]. Tirone et al. reported that high total cholesterol levels do not affect implant success but negatively affect graft healing [13].

This study began when we questioned the effect of atorvastatin on bone metabolism in the literature [5]. In the drug-user group, the dose was determined to be 10 mg/kg atorvastatin. Statins generally undergo high intestinal and/or hepatic first-pass elimination, which results in a short half-life. Because cholesterol synthesis is greater at night, statins with a short plasma half-life provide greater cholesterol reduction when administered at night. However, those with a long half-life (atorvastatin and rosuvastatin) may be equally effective when administered in the morning [14]. Compared with milligram equivalent doses of simvastatin, pravastatin, lovastatin, and fluvastatin, as well as a normal diet, 10, 20, and 40 mg of atorvastatin have been found to provide a greater reduction in LDL and total cholesterol [14]. Nicholls et al. found no difference between the use of 5 mg/kg atorvastatin in hyperlipidemic rabbits and the total cholesterol and triglyceride levels in drug-free groups [15]. Rajamanann et al. reported that in rabbits with hypercholesterolemia, the use of 3 mg/kg atorvastatin for 8 weeks resulted in a decrease in total cholesterol but did not result in a significant decrease [16]. Based on this information, we decided to use atorvastatin 10 mg/kg per day to lower hypercholesterolemia in this study. To the best of our knowledge, the effect of this dose on graft healing in mandibular bone defects has not been investigated. İlker et al. thought that local application of statins in mandibular bone defects was more effective, and applied simvastatin at 2.5 mg/ml concentration to a collagen gelatin sponge in their study. As a result of the study, they stated that they found the local application effective [17]. Taşdemir et al. found similar results with simvastatin-mixed autogenous bone grafts and xenografts when the autogenous graft was mixed and applied with simvastatin [18].

Gautam et al. stated that the application of rosuvastatin gel, which was obtained using appropriate techniques for intraosseous defects, together with bone grafts, contributes to clinical improvement [19]. Some researchers have suggested that the positive effects of lipid-lowering drugs such as statins on bone metabolism may also occur during osteogenesis around implants [1]. Yaghobee et al. stated in their clinical study that there was no significant difference between mixing and not mixing simvastatin in xenografts in maxillary sinus procedures [20].

To the best of our knowledge, no studies have been conducted on the local application of atorvastatin in either maxillary or mandibular defects. We agree that local application will be more effective than systemic application, but it is unclear to what extent the material will absorb the drug in particulate grafts, such as xenografts. We believe that materials, such as xenograft, autograft, and allograft, which are not sponge-shaped, will not give realistic results in local drug application, due to the concern that even if applied locally to the area, it will not show the desired effect by spreading to the surrounding tissues.

We believe that the first reason for the effectiveness of atorvastatin is the decrease in blood lipid levels, which do not impair the healing mechanism, and the second reason is its effect on angiogenesis. Currently, atorvastatin increases angiogenesis by affecting mesenchymal stem cells [21]. The second and most important stage of graft healing is revascularization. During this period, new vessels move around the graft and provide nutrition during the healing process [22, 23]

In this study, we confirmed that drug use in hyperlipidemia is important for graft regeneration and that high blood lipid levels negatively affect bone healing. Statins are effective drugs not only for cardiovascular health but also for graft healing metabolism in oral implantology. We can say that it is necessary to pay attention to the choice of surgical method and material, considering the risks that may be encountered in cases of questioning the use of drugs in patients with high blood lipid levels and not using them.

Bone regeneration occurs spontaneously in a favorable environment with a good blood supply and mesenchymal cells. However, critical-sized defects cannot be completely healed owing to a lack of mechanical support. Under these conditions, additional materials such as grafts are required to aid in bone regeneration [24]. During the healing process, a barrier membrane is applied between the graft and soft tissue to prevent epithelial and connective tissue cells from settling in the defect area and to activate osteogenic cells in the bone walls of the defect. This surgical technique is called guided bone regeneration (GBR) [25]. In this study, xenografts were used for the regeneration of critical-sized defects, and a collagen membrane was used as the soft tissue barrier. There were no complications, such as wound dehiscence or secondary infection, during the healing process. While the tissues were removed for examination, no complications, such as soft tissue collapse, graft disintegration, and penetration into the surrounding tissue, were observed.

Histomorphometric methods were used for new bone area measurements, and both H&E and MT staining methods were used. The new bone area was found to be clinically and statistically significantly higher in the atorvastatin group. TRAP was used to determine osteoclast activity in the defect area and AP staining was used to determine osteoblast activity. TRAP activity is considered an important cytochemical marker of osteoclasts; its concentration in the serum is used as a biochemical marker of osteoclast function and the extent of bone resorption [26]. AP levels are higher in the lacunae and periosteal surfaces in areas of active bone mineralization. AP activity is evident around new bones [27]. Quantitative measurement of osteoclastogenesis and osteoblastogenesis can be performed by analyzing TRAP and AP activities [28, 29]. Dolci et al. stated that atorvastatin inhibited osteoclasts and significantly reduced the number of TRAP-positive cells [30]. However, there have been no studies on osteoblast activity. We also observed osteoblast activity in our study, and AP immunoreactivity was higher in the atorvastatin group, which was consistent with the new bone area, whereas TRAP immunoreactivity was higher in the drug-free control group.

In this study, the rabbit species with which we could best follow the hyperlipidemia model and mandibular graft healing were used. Pecoraro et al. suggested that rabbits are the most appropriate animal species for studying the effects of statins on lipids [3]. In addition, the embryological development of rabbits is similar to that of humans. The mandibular premolar/molar region had a width of 17 mm, height of 16 mm, and depth of 6 mm. This region is suitable for biomaterial analysis [31, 32]. Shaha et al. defined a defect formed up to the tooth roots with a diameter of 10 mm in the rabbit corpus as a partial-thickness defect. They defined the defect with a diameter of 10 mm, in which the buccal and lingual cortex were completely removed, as a full-thickness defect and stated that this defect did not heal spontaneously [32]. The defect size was determined as 10 mm in diameter and 5 mm in depth. Regenerative stem cell sources in the periodontal ligament were eliminated by extracting the teeth and roots in the region. For the defect, removal of the buccal cortex and preservation of the lingual cortex were preferred. Therefore, we aimed to minimize the probability of graft disintegration during the recovery period.

The lack of human studies is a limitation of this study, although it has been conducted in rabbits, which show the closest recovery to humans. It is unclear whether the angiogenic effect of atorvastatin increases healing and whether there is a difference between the effect of locally applied atorvastatin and systemic application in defect healing. In addition, regardless of the type of statin used, the effect of mixing local applications in the use of grafts is not available in the literature. There is a need for multicenter clinical studies with larger sample sizes that address all of these issues.

## Conclusion

Clinicians should be aware of the selection of surgical methods and materials, considering the importance of questioning the use of drugs in their patients, and considering the risks in cases, where they are not used in patients with hyperlipidemia.

## Data Availability

The data sets created and analyzed during the current study are available from the corresponding author upon request.

## Abbreviations

TG: Triglyceride
LDL: Low-density lipoprotein
HDL: High-density lipoprotein
BMP: Bone morphogenetic proteins
GBR: Guided bone regenerations
MT: Masson’s trichrome
H&E: Hematoxylin and eosin
PBS: Phosphate buffer
TRAP: Tartrate-resistant acid phosphatase
AP: Alkaline phosphatase
VLDL: Very low-density lipoprotein
